# Successful Management of an Inadvertent Placement of a Nephrostomy Tube Into the Inferior Vena Cava Following Percutaneous Nephrolithotomy: A Case Discussion and Literature Review of a Rare Complication

**DOI:** 10.7759/cureus.44422

**Published:** 2023-08-31

**Authors:** Hesham H AbdelAziz, Mohamed H Gad

**Affiliations:** 1 Department of Urology, Al Soliman Hospital, Port Said, EGY; 2 Department of Urology, Guy's and St. Thomas' National Health Service (NHS) Foundation Trust, London, GBR

**Keywords:** embolisation, arteriovenous pseudo-aneurysm, nephrostomy tube, inferior vena cava, percutaneous nephrolithotomy

## Abstract

In a percutaneous nephrolithotomy (PCNL) procedure, the placement of the nephrostomy tube is usually inserted last to monitor and maintain urine drainage, avoid potential urine extravasation, and ensure hemostasis. In this report, we provide a clinical case involving the misplacement of a nephrostomy tube, resulting in direct perforation of the inferior vena cava (IVC) after undergoing one-sided PCNL that was successfully treated conservatively, and investigate the current management censuses from the literature for intravenous misplacement of a nephrostomy tube. In our patient, the tip of the nephrostomy catheter was located in the IVC. It was successfully managed using a one-step catheter withdrawal with the surgical vascular team on standby for any potential encounters with massive uncontrollable bleeding. An enhanced CT angiogram on day 14 post-PCNL revealed a lower polar renal arteriovenous pseudoaneurysm which required our patient to undergo selective angioembolization, resulting in maximal parenchymal preservation. The patient was successfully managed and discharged uneventfully. Thirteen cases that have reported inadvertent misplacements in the PubMed database have been discussed in this review. Our case would be the first documented report where a percutaneous nephrostomy drainage tube pierced through the IVC directly.

Our case provides an argument for patients to be managed by tube withdrawal under one-step fluoroscopic guidance. Intensive care measures and ultrasound monitoring for two hours followed by another CT angiogram proved effective successful conservative management in a high-volume urologic practice.

## Introduction

Percutaneous nephrolithotomy (PCNL) is considered to be the standard therapeutic approach to patients with large complex ureteral/renal calculi when neither ureteroscopy (URS) nor shock wave lithotripsy (SWL) would likely accomplish stone clearance.

Since the introduction of percutaneous nephrostomy under radiological control by Fernström and Johansson in 1976, PCNL has been the first-line method for the treatment of upper urinary calculi, with it being both a feasible and effective method in the treatment of medium and large stones, with the advantages of removing large stone burdens in a single setting as compared to the need for multiple surgeries with extracorporeal SWL or URS leading to less trauma, fewer hospital stays, and quicker recovery [1,2]. However, potential complications associated with PCNL would include bleeding (with a transfusion rate of 1% to 10%), vascular injuries (0.5%), pleural injuries (1% to 10% of upper pole tracts), and infections [3-5].

Inadvertent insertion of a percutaneous nephrostomy drainage tube into the inferior vena cava (IVC) can lead to intraoperative vascular injury and postoperative hemorrhage, making hemorrhage the most notable complication of PCNL with transfusion rates needed in up to 10% of all cases [3-5]. In this study, we report a case of an IVC injury and secondary thrombosis after PCNL. We also discuss the current reports in the literature on managing such complications.

This article was previously presented as a meeting abstract at the European Association of Urology (EAU) UROtech: a joint meeting of the EAU Section for Uro-Technology and the EAU Section of Urolithiasis in collaboration with the EAU Robotic Urology Section on May 26-28, 2022.

## Case presentation

A 45-year-old female with a background of recurrent staghorn stones of the right kidney, ischemic heart disease, and hypertension, was admitted with the main complaint of recurrent persistent right flank pain. A CT urogram revealed a staghorn renal calculus with resultant hydronephrosis. A PCNL was planned for access through the inferior calyceal tract with ultrasonic lithotripsy and fragment extraction.

From the prone position, a brisk amount of venous bleeding was observed when we fixated the nephrostomy tube to the skin. Fluoroscopy showed the extrarenal misplacement of the nephrostomy tube. The 24 Fr nephrostomy was then clamped to attempt to achieve hemostasis. Ultrasonography on an ambulatory basis revealed the tip of the tube inside the IVC. The patient was ambulatory and transferred to the radiology department within 30 minutes after the ultrasound examination to get an enhanced contrast CT, which revealed the presence of the nephrostomy tube inside the vena cava with no collection of blood around the tube. An urgent emergency in-hospital consultation with the surgical team was performed to take the tube off in one step and to intervene and operate an emergency open surgery in the event of uncontrolled bleeding. Based on the vascular consultant’s advice, the nephrostomy tube was completely pulled off under fluoroscopic guidance. The surgical team was on standby for any potential encounters with massive uncontrollable bleeding and to follow up with the patient for up to two hours such events of blood transfusion, angiography, or even angioembolization to control any potential hematuria. Fluid resuscitation and replacement, antimicrobial treatment, anticoagulation treatment, and adequate bed rest were undertaken.

**Figure 1:**
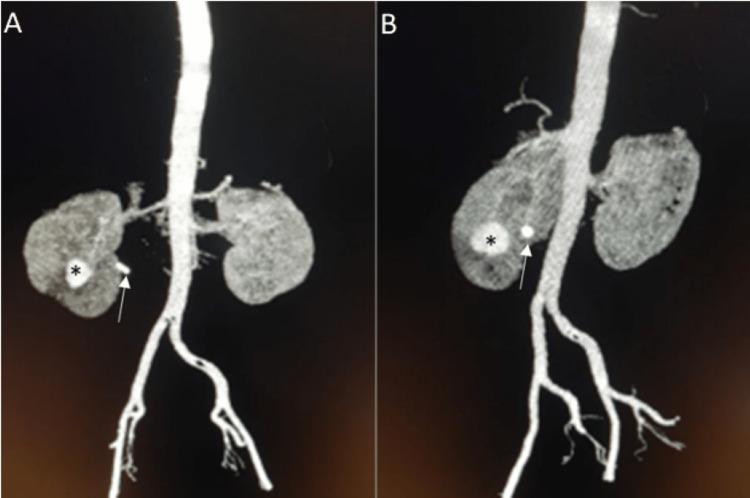
A two-week follow-up multidetector CT, coronal plane maximum intensity projection reconstruction demonstrates renal arteriovenous fistula (arrow) with associated pseudoaneurysm

On day 14 post-PCNL, the patient was presented with macroscopic hematuria and clot retention, suggesting acute urine retention. A multidetector CT angiogram scan revealed a slightly hyperdense lesion on the right lower polar of the kidney showing intense enhancement in the arterial phases, suggestive of lower polar renal arteriovenous pseudoaneurysm, measuring about 3 x 2.5 x 2.4 cm (Figure 1). The scan also showed mild hydroureteronephrosis down to a stone seen at the right lumbar ureter with a small lower polar perinephric collection measuring about 2 x 2.3 cm. The residual of the staghorn calculus of the right kidney, about 1.5 cm (about 0.59 in) in the pelvic-ureteric junction, was displaced and retracted back to the renal pelvis, and a double J stent was indwelled on day 16 to ensure urinary flow into the urinary tracts and to adequately drain the collecting system.

The patient was then transferred on that same day of stenting to another specialized tertiary center for angioembolization. Selective lower polar arterial embolization was carried out and since then has been showing smooth gradual recovery of their general status.

## Discussion

Intravenous nephrostomy tube misplacement is an uncommon PCNL complication, with an incidence rate of 0.02 [6]. Few articles have reported the inadvertent insertion of a nephrostomy tube in the renal vein, IVC, or the atrium, and even fewer have reported recurrence of thrombosis [3,4,6-12]. To date, only 13 cases have been reported in the PubMed database, and all the nephrostomy tubes were either pierced through the ipsilateral renal vein or through the IVC. One case reported a misplacement into the atrium and one misplacement into the contralateral renal vein [6].

Hemorrhage is the most significant complication of PCNL, with acute hemorrhage due to injury to the main renal vessels or IVC, which is uncommon and occurs in less than 0.5% of cases and with blood transfusion rates in the literature ranging from 0.5% up to 23% [3,11].

Several case reports in the open literature have published their complicated hemorrhage due to the misplaced percutaneous nephrostomy tube into a major vessel after PCNL. The data from these publications are summarized in Table 1.

**Table 1:** Published reports of intravenous misplacement of nephrostomy tubes IVC: inferior vena cava, PCNL: percutaneous nephrolithotomy, DVT: deep vein thrombosis

Author/year	Age	Sex	Affected side	Catheter type	Operation	Location of misplacement	Delayed finding	Withdrawal steps	Antithrombotic therapy given
Mazzucchi et al., 2009 [3]	35	Female	Left	Nephrostomy tube	PCNL	Renal vein, IVC	Yes	Removed two-step under fluoroscopy	No
Mazzuchi et al., 2009 [3]	52	Male	Left	Nephrostomy tube	PCNL	Renal vein	Yes	Removed one-step under fluoroscopy	No
Kotb et al., 2013 [4]	50	Male	Left	Foley	Percutaneous nephrostomy	Renal vein, IVC	Yes	Removed one-step open pyelotomy	No (patient developed DVT as a result)
Chen et al., 2014 [6]	38	Female	Left	Nephrostomy tube	PCNL	Renal vein, IVC	No	Two-step under fluoroscopy	No
Chen et al., 2014 [6]	42	Male	Left	Nephrostomy tube	PCNL	Renal vein, IVC	Yes	Two-step under CT monitoring	No
Chen et al., 2014 [6]	48	Male	Left	Nephrostomy tube	PCNL	Renal vein	Yes	Removed one-step under ultrasound	No
Dias-Filho et al., 2005 [7]	63	Female	Left	Foley	Catheter placement	Renal vein, IVC, right atrium	Yes	One-step under fluoroscopy	No
Fu et al., 2017 [8]	26	Male	Left	Nephrostomy tube	PCNL	Renal vein, IVC	Yes	Removed one-step via open pyelotomy	No
Fu et al., 2017 [8]	68	Male	Right	Nephrostomy tube	PCNL	Renal vein	Yes	Removed one-step via open pyelotomy	No
Li et al., 2013 [9]	32	Female	Left	Nephrostomy tube	PCNL	Renal vein, IVC	Yes	Removed two-step under ultrasound	Yes
Shaw et al., 2005 [10]	54	Male	Right	14F Foley catheter, nephrostomy tube	PCNL	Renal vein	No	Two-step under fluoroscopy	Yes
Wang et al., 2013 [11]	66	Female	Left	Nephrostomy tube	PCNL	Renal vein	Yes	One-step under fluoroscopy	No
Ge et al., 2020[12]	31	Female	Right	Nephrostomy tube	PCNL	Right renal vein, IVC, left renal vein	Yes	Two-step under CT	Yes

Mechanisms of misplacement

According to current literature, there are various factors that may contribute to rare complications such as intravenous tube misplacement. In particular, three possible mechanisms of misplacement have been identified and are interrelated. Chen et al. [6] proposed the first mechanism of misplacement, which was further explained by Fu et al. [8]. In their cases, it is believed that the dilator sheath punctured through the renal parenchyma and into the renal vein, which then led to perforation of the vein during the subsequent dilatation guided by the wire.

The second possible mechanism of intravenous tube misplacement has been proposed by Mazzucchi et al. [3] and Wang et al. [11]. They reported that the renal vein could be perforated by instruments used during PCNL or that the proximity of the injured vein to the Amplatz sheath could have inadvertently pierced the nephrostomy tube into the venous vessels.

The third misplacement manner can be observed in catheter placement as revealed by Dias-Filho et al. and Kotb et al. The proximity of the renal pelvis and major posterior calyces to the renal vein potentiates the risk of injury and consequently predisposes the risk of venous trunk perforation by the guidewire and dilation when the tube is placed inside the vessel directly when radiological guidance is not used [7]. In our case, the passage of the nephrostomy draining tube directly into the IVC was due to blind guidewire manipulation that subsequently caused blind dilatation of the venous tract. The literature proposed that the synergistic use of fluoroscopy and sonography might contribute to better visualization and enhanced localization of the kidneys in three planes, saving the drawbacks of administrating contrast material to patients with potential rental failure, eliminating the uncertainty associated with needle insertion and kidney depth from fluoroscopy's single visual plane, and, thus, reducing the likelihood of multiple punctures and parenchymal damage [13].

Intraoperative risk factors from the literature

The formation of multiple renal pelvicalyceal punctures and access tracts, stone burden, and prolonged operative time have all been identified as some of the main predictors of post-PCNL bleeding [14-16], while Srivastava et al. concluded that stone size is a significant predictor in resultant hemorrhage post-PCNL [17].

Turna et al. [15] did concur with Akman et al. [14], adding further that partial and complete staghorn stones are more vulnerable to bleeding because they are maneuver-challenging and may increase the number of accesses needed to completely clear the pelvicalyceal system from stone, hence increasing the risk of parenchymal and pelvicalyceal perforation, which can lead to bleeding.

Some case reports have described a noninvasive method to address the misplacement of nephrostomy tubes into major vessels during PCNL. This method involves clamping the catheter and removing and repositioning the tube into the collecting system with the help of guided fluoroscopy [6]. Fu et al. [8] used this approach and performed open surgery to manage the intravenous misplacement, gradually withdrawing the tube under fluoroscopic guidance.

According to current literature, controlling hemorrhage caused by intravenous misplacement during PCNL can be achieved using a nephrostomy catheter. Even when there is perforation into the major renal vein, the catheter can be used to help the tract heal by gradually withdrawing it in stages with the guidance of fluoroscopy. Chen et al. reported three cases of intravenous misplacement after PCNL, where the tip of the tube was in the IVC in two cases and in the renal vein in one case. All cases were successfully managed using a one-step or two-step tube withdrawal method while under close monitoring [6].

In contrast to the approach taken by Fu et al. and Chen et al., Zahrani et al. [18] reported a case where a nephrostomy tube was misplaced into the IVC of a patient with obstructive uropathy in their solitary kidney. They removed the misplaced catheter in one step while the patient was under general anesthesia. They used the percutaneous intravenous balloon tamponade technique and alternated the patient's position between prone and supine during the procedure [18]. In the current case being discussed, an immediate CT angiogram showed that the nephrostomy tube was inside the vena cava with no collection around it. Based on the advice of a vascular consultant, the tube was completely withdrawn under fluoroscopy guidance.

Mazzucchi et al. [8] reported two cases of misplaced nephrostomy catheters into the ipsilateral renal vein and IVC. These cases occurred at the end of PCNL, and the nephrostomy tubes were removed with consultation from the surgical team standing by to intervene and perform an emergency open surgery in case of uncontrolled bleeding. However, no bleeding occurred. The authors attributed the mishap to the proximity of the Amplatz sheath to the injured venous site, which could have inadvertently directed the nephrostomy tube into the IVC.

Kotb et al. [4] reported a case of inadvertent insertion of a percutaneous silicon catheter into the IVC following PCNL. Through open surgical pyelotomy, the nephrostomy tube was pulled back into the renal pelvis under fluoroscopic monitoring, resulting in no hemorrhage but thrombosis was formed [4].

Dias-Filho et al. reported a case of misplaced catheterization into the IVC, with the nephrostomy catheter migrating into the right atrium. In their report, while changing the nephrostomy tube, they passed a guide wire under no fluoroscopic monitoring that dilated the tract, before inserting a new silicon catheter. They concluded that the perforation caused by passing the guidewire blindly, with dilatation of the injured venous tract, resulted in the catheter perforating to the IVC [7].

Chen et al. [6] reported three cases of intravenous misplaced nephrostomy draining tubes. In two of these cases, the nephrostomy catheters were inadvertently dislodged into the IVC and were removed under the guidance of ultrasound. In the third case, the catheter was perforated into the renal vein and was removed under close monitoring with the surgical team ready to intervene if uncontrollable bleeding occurred [6]. The management strategy for these cases differed depending on the depth of tube penetration. In one case, the tube was immediately withdrawn, while in the other two cases, the tube was withdrawn into the renal pelvis and removed if the patient was hemodynamically stable and under close monitoring. None of the patients received antithrombotic therapy nor developed thromboembolic events. In addition to strict bed rest and intravenous antibiotics, the authors recommended immediately closing the tube once intravenous misplacement is discovered and repositioning the tube at the site of entry into the renal vein, trunk, IVC, or atrium under CT, ultrasound, or fluoroscopic monitoring [6].

Use of antegrade pyelography

Following PCNL, a nephrostomy tube is typically placed in the collecting system to help achieve hemostasis and prevent urine extravasation, which could lead to the formation of a urinoma [16]. If severe venous bleeding occurs during the stone removal procedure, it is advisable to pause the procedure and temporarily close the inserted nephrostomy tube to allow for natural hemostasis to occur within the kidney. In such a complication, early detection using antegrade pyelography can provide valuable information about the success of stone removal, the presence of any residual stones or fragments, and the evaluation of ureteral integrity. Of the 13 patients identified in this review, 11 patients had delayed detection. For our patient, even though early detection of the misplaced percutaneous tube in the operating table did allow an early withdrawal option, that did not spare further complications later postoperatively.

Pseudoaneurysm formations and arteriovenous fistulas

When bleeding is uncontrollable and natural hemostasis is not possible, renal angiography and artery embolism can be resorted to [19]. Venous injuries causing bleeding during PCNL can be classified as immediate, early, or delayed. Early injuries are seen two to three days postoperatively maybe because of pseudoaneurysm formation or arteriovenous fistula. Immediate injuries are usually managed by immediately clamping nephrostomy tubes to allow clot formation and subsequent dissolution. Immediate injuries can be halted due to the venous resiliency and the elasticity of the intrarenal venous system within the kidney, whereas artery injury that may induce severe bleeding requires angioembolization.

Ultrasound and contrast CT can be used for the initial evaluation, but digital subtraction angiography (DSA) is the most reliable diagnostic tool. In cases of hemodynamically unstable patients with tachycardia, gross hematuria, low serum hemoglobin levels (<10 g/dL), blood transfusions, and hypotension, DSA should be considered the gold standard [19]. Arterial injuries require immediate surgical intervention through selective or super-selective angioembolization, which has a high success rate in stopping severe bleeding [20]. Wang et al. reported arteriovenous pseudoaneurysm formation as a result of intravenous nephrostomy tube misplacement. In their case, the nephrostomy tube was withdrawn in stages with a standby vascular team in case of uncontrolled bleeding. The pseudoaneurysm was treated with percutaneous embolization uneventful [11].

When looking at the current literature, regardless of whether managing open or endovascular, the current recommendations to manage severe hemorrhage is to first insert an IVC filter to prevent dislodgement of the thrombus, and then the thrombus can then be aspirated by endovascular luminal aspiration or removing the thrombus through open surgery. An advantage of the endovascular method is that it is minimally invasive, but a disadvantage is that it is difficult to remove the thrombus when it is large or even growing. An advantage of an open surgery would be the efficiency in removing any thrombus as seen, but it being more invasive makes it less favorable.

## Conclusions

Intravenous nephrostomy tube misplacement is a rare PCNL complication. Our case provides a compelling argument for patients to be managed by tube withdrawal under one-step fluoroscopic guidance. Intensive care measures and ultrasound monitoring for two hours followed by another CT angiogram proved effective successful conservative management in high-volume urologic practice. Our case highlights the importance of prompt diagnosis of major venous perforation and demonstrates that this can be managed conservatively by the catheter being withdrawn in stages or, according to the recent recommendations in the literature, using a tamponade nephrostomy catheter. One disadvantage of one-step tube withdrawal is the increased risk of developing pseudoaneurysm and/or arteriovenous fistula, especially in recurrent non-stented PCNL.
